# Antimicrobial Photodynamic Therapy for Superficial, Skin, and Mucosal Fungal Infections: An Update

**DOI:** 10.3390/microorganisms13061406

**Published:** 2025-06-17

**Authors:** Laura Beatriz Borim da Silva, Ivana Giovannetti Castilho, Fabiana Alves de Souza Silva, Mahmoud Ghannoum, Maíra Terra Garcia, Paulo Henrique Fonseca do Carmo

**Affiliations:** 1 Departamento de Genética, Microbiologia e Imunologia, Instituto de Biociências de Botucatu, Universidade Estadual Paulista (UNESP), Botucatu 18618-689, SP, Brazil; 2 Departamento de Biociências e Diagnóstico Bucal, Instituto de Ciência e Tecnologia, Universidade Estadual Paulista (UNESP), São José dos Campos 12245-000, SP, Brazil; 3 Center for Medical Mycology, Department of Dermatology, Case Western Reserve University, Cleveland, OH 44106, USA

**Keywords:** photodynamic therapy, photosensitizer, dermatophytes, *Candida* spp., mycoses, antifungal

## Abstract

The global burden of fungal infections is rising at an alarming rate, with superficial, cutaneous, and mucosal mycoses among the most prevalent. Conventional treatments rely on oral and topical antifungal agents; however, these therapies are often limited by adverse effects, toxicity, frequent recurrence, and poor patient adherence due to prolonged treatment regimens. Moreover, the emergence of antifungal resistance and multidrug-resistant species such as *Candidozyma auris* and *Trichophyton indotineae* highlights the urgent need for alternative therapeutic strategies, such as antimicrobial photodynamic therapy (aPDT). aPDT is based on photophysical and photochemical processes involving a photosensitizer (PS), a light source, and molecular oxygen. When combined, these elements generate reactive oxygen species that selectively destroy microbial cells. In this review, we explore various PSs and their effectiveness in aPDT against infections caused by dermatophytes, *Candida* spp., and other pathogenic fungi. Promisingly, aPDT has demonstrated antifungal activity against both susceptible and resistant strains. In addition, aPDT has been successfully used in cases of mycoses unresponsive to conventional therapies, showing favorable clinical outcomes and overall safety. Current evidence supports aPDT as a valuable strategy for the management of cutaneous, mucosal, and superficial fungal infections and as a potential strategy to combat antifungal resistance.

## 1. Introduction

The global burden of fungal infections is increasing at an alarming rate, with a recent estimate of 6.5 million annual cases and 3.8 million associated deaths worldwide [1]. In this context, superficial, cutaneous, and mucosal mycoses are highly prevalent, accounting for 1.1 million cases [2]. In the U.S., Benedict et al. [3] conservatively estimated the economic burden of fungal infections at USD 11.5 billion in 2019, with at least USD 3.7 billion (approximately 32.17%) allocated to managing superficial, cutaneous, and/or mucosal mycoses. Furthermore, ongoing wars and global warming are expected to significantly contribute to the increased occurrence of these diseases in several countries [4,5].

Superficial mycoses are infections restricted to the outer layers of the skin and its appendages, including hair and nails. These infections are primarily caused by *Malassezia* spp., *Hortaea werneckii*, *Trichosporon* spp., and *Piedraia hortae* [6,7,8,9]. In addition, dermatophytes from the genera *Trichophyton*, *Epidermophyton*, *Microsporum,* and *Nannizzia*, as well as *Candida* spp. and related species, are responsible for onychomycosis, fungal infections that affect the nails [10,11]. Superficial fungal infections are more prevalent in tropical and subtropical regions, where heat, humidity, and other favorable conditions contribute to their development and persistence [12].

Cutaneous and mucosal mycoses are highly prevalent infections worldwide, affecting the mucosa of the gastrointestinal and genitourinary tracts, as well as the deeper layers of the skin [3,13]. These infections are of significant concern due to their morbidity, high transmission potential, and impacts on patients’ quality of life [3,14]. While dermatophytes, *Candida* spp., and related species are the primary causative agents, filamentous fungi such as *Curvularia* spp. and *Aspergillus* spp. have also been associated with rare cases of skin mycoses [15,16].

Conventional therapy for superficial, skin, and mucosal mycoses involves the use of oral and topical antifungal agents, including itraconazole, terbinafine, ketoconazole, fluconazole, nystatin, ciclopirox olamine, and amorolfine [12,17,18]. However, treatment with these antifungals faces several limitations, such as adverse effects, hepatotoxicity, high costs, recurrence, and poor adherence due to prolonged treatment regimens [19]. In addition, antifungal resistance and the emergence of multidrug-resistant species further restrict the already limited antifungal arsenal [20,21,22]. Therefore, the development of new therapeutic strategies against these infections is highly desirable. In this context, antimicrobial photodynamic therapy (aPDT) has gained attention due to its well-established biological effects, low toxicity, and non-invasive nature [23,24,25]. In this review, we discuss recent advancements in aPDT strategies for treating superficial, cutaneous, and mucosal fungal infections. We also provided an overview of both well-established and emerging PSs, highlighting their effects in in vitro and in vivo models.

## 2. PDT and aPDT

Photodynamic therapy (PDT) is an approach based on photophysical and photochemical reactions that lead to selective destruction of target cells through the generation of reactive oxygen species (ROS) [25,26]. Initially popularized in oncology, PDT has been used as a therapeutic approach for a wide range of cancers, offering several advantages, including low systemic toxicity and fertility preservation [27,28]. In microbiology, PDT-based strategies have been used for over a century [29]. However, the growing concern over antimicrobial resistance strains has enhanced the interest in PDT as an alternative antimicrobial treatment—an approach known as aPDT [30,31,32]. Both PDT and aPDT share the same three essential components: a PS, a light source, and molecular oxygen [25,33].

PSs are photoactive molecules that play a critical role in the efficiency of PDT/aPDT. After administration, PSs penetrate and accumulate in the target cells during a period known as the pre-irradiation time (PIT). The duration of the PIT depends on several factors, including the type of PS, the characteristics of the irradiated tissue, and the nature of the target [34,35]. Following this period, the PS must be activated by exposure to a light source with a wavelength appropriate for its excitation. Currently, two main types of light sources are used in PDT and aPDT: lasers and light-emitting diodes (LEDs) [25,36]. Lasers emit monochromatic, coherent light, which allows for high precision in targeting and deeper tissue penetration. However, they operate within a narrow wavelength range, are expensive, and often require optical systems to broaden the beam for larger treatment areas. In contrast, LEDs emit incoherent light across a broader wavelength spectrum, making them a more versatile and cost-effective option. In addition, LEDs offer advantages such as ease of use, lower energy consumption, and broader illumination coverage [37,38].

**Figure 1:**
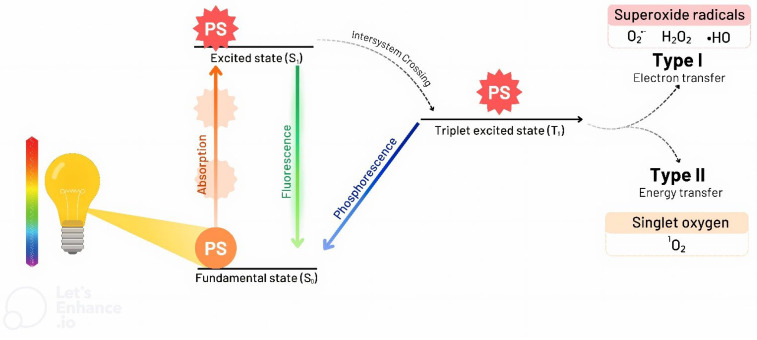
**Simplified Jablonski diagram illustrating the processes involved in photodynamic therapy (PDT).** The process begins with the activation of the photosensitizer (PS) through irradiation using a light source with an appropriate wavelength. In its ground state (S_0_), the PS absorbs light energy and transitions to an excited singlet state (S_1_). From the S_1_ state, the PS can either return to the S_0_ state by emitting fluorescence or undergo intersystem crossing to the more stable triplet excited state (T_1_). Once in the T_1_ state, the PS may return to the S_0_ state via phosphorescence or initiate photodynamic reactions by transferring excess energy to molecular oxygen. Two main reaction pathways can occur. The Type I reactions involve electron or hydrogen transfer from the excited PS to surrounding biomolecules, generating reactive oxygen species (ROS) such as superoxide anion (O_2_^−^), hydrogen peroxide (H_2_O_2_), and hydroxyl radical (OH^−^). The Type II reactions involve direct energy transfer from the PS to molecular oxygen, producing singlet oxygen (^1^O_2_), a highly reactive species that causes oxidative damage to cellular structures.

Upon exposure to a light source, the PS absorbs light energy, transitioning from its ground state (S_0_) to an excited singlet state (S_1_). The excited S_1_ state is unstable and may follow one of two pathways: it can return to the S_0_ state by emitting fluorescence or undergo intersystem crossing to a more stable triplet excited state (T_1_). Once in the T_1_ state, the PS may either return to the S_0_ state via phosphorescence or engage in two main photophysical pathways in the presence of molecular oxygen—Type I and Type II reactions [28,38] (Figure 1).

In Type I reactions, the excited PS interacts directly with biomolecules such as lipids, proteins, and nucleic acids through hydrogen abstraction or electron transfer. This leads to the generation of free radicals, which, in the presence of molecular oxygen, form ROS such as superoxide anions (O_2_^−•^), hydroxyl radicals (HO^•^), and hydrogen peroxide (H_2_O_2_). In Type II reactions, energy from the T_1_ state is transferred directly to molecular oxygen, producing singlet oxygen (^1^O_2_), a highly reactive oxygen species capable of causing oxidative damage to cellular components. Both pathways ultimately result in oxidative stress and cell death, which form the basis of the cytotoxic effects seen in PDT and aPDT [25,38,39].

These reactions are possible because the T_1_ state of the PS is more stable and longer-lived, allowing efficient energy transfer (electrons) to molecular oxygen and/or surrounding biomolecules [35]. The availability of molecular oxygen is the third critical component influencing the efficacy of PDT/aPDT. Molecular oxygen interacts with the PS in its T_1_ stage, leading to the generation of ROS. Importantly, ROS production is proportional to the availability of molecular oxygen, and hypoxic conditions can significantly reduce oxidative burst and treatment effectiveness [40]. Although both Type I and Type II reactions may occur simultaneously, Type II reactions are typically associated with greater cytotoxicity and are considered the primary contributors to the therapeutic effects of PDT and aPDT [25].

While oxygen is essential for both Type I and Type II reactions, oxygen-independent photodynamic strategies have also been explored. In this alternative mechanism, classified as Type III reactions, certain PSs, such as psoralen and tetracycline, directly transfer electrons to biomolecules, leading to the production of reactive inorganic radicals. This oxygen-independent mechanism holds potential for treating anaerobic infections, hypoxic tissues, and dense biofilms [41,42,43]. However, further studies are necessary to elucidate the exact mechanisms by which Type III reactions exert their effects on microbial cells and to validate their clinical applicability.

Several studies have investigated the impact of aPDT on pathogenic fungi. The oxidative stress resulting from increased ROS formation following aPDT leads to the disruption of vital fungal components, including the cell membrane, proteins, and nucleic acids. This multi-target mechanism significantly reduces the likelihood of resistance development, making aPDT an effective, targeted, and safe therapeutic strategy [44].

Regardless of these advantages, a major concern lies in the structural similarities between fungal and mammalian cells, as both are eukaryotic cells [42,45]. This raises potential issues of host toxicity. Therefore, careful selection of the PS is critical for ensuring therapeutic safety and efficacy in treating fungal infections. The ideal PS should meet the following criteria: (a) high absorption efficiency—cationic PSs exhibit better interaction with negatively charged microbial membranes than neutral or anionic compounds, (b) favorable photochemical properties—including a high quantum yield of ROS, especially single oxygen and long-lived triplet state (T_1_), (c) appropriate light activation range—wavelengths between 400 to 800 nm are optimal to ensure tissue penetration and therapeutic efficacy while minimizing adverse effects, (d) low dark toxicity—the PS should remain non-toxic in the absence of light and exhibit minimal phototoxicity upon ambient light exposure, (e) high selectivity—preferential accumulation in fungal cells over host cells, and (f) practicality—ease of synthesis, formulation stability, and low production cost [42,43,46].

Despite these challenges, aPDT continues to gain attention as a promising adjunct to alternative treatment for fungal infections. Numerous studies have demonstrated its effectiveness against a broad range of fungal pathogens [45,47,48].

### 2.1. aPDT for the Treatment of Dermatophytosis

Dermatophytosis is a common cutaneous mycosis and the most prevalent human skin infection worldwide, with an estimated incidence ranging from 20% to 25% among immunocompetent individuals [49,50]. In the U.S., the economic impact of dermatophytosis is substantial. In 2019, the total economic burden—which included direct medical costs, productivity losses due to work absences, and premature mortality—was estimated to exceed USD 1.1 billion [3].

These infections were caused by dermatophytes, a group of keratinophilic fungi with global distribution that primarily infect keratinized tissues such as skin, nails, and hair [51,52]. Dermatophytes are capable of surviving in soil and colonizing animals [53], which allows infections to be caused by zoophilic, geophilic, and anthropophilic species. While seven genera encompass dermatophytes [54], species from the genera *Trichophyton*, *Epidermophyton*, *Nannizzia*, and *Microsporum* are commonly associated with human and animal infections [49,52].

Conventional treatment of dermatophytosis is based on topical and oral antifungals such as terbinafine, ketoconazole, itraconazole, griseofulvin, amorolfine, and ciclopirox olamine [55]. However, prolonged use—especially of oral antifungals—has been linked to adverse effects, including hepatotoxicity. Moreover, conventional therapies are often associated with high relapse rates, which can compromise patient adherence due to lengthy treatment regimens and recurring manifestations [51,56]. In addition, the recent rise of multidrug-resistant species such as *Trichophyton indotineae* [20,21] highlights the need for alternative therapy strategies such as aPDT. This section reviews the potential of various PSs—including methylene blue, 5-aminolevulinic acid, and hypericin, among others—for use in aPDT against dermatophyte infections (Table 1).

#### 2.1.1. Methylene Blue

Methylene blue (MB) is an aromatic heterocyclic basic dye with high water solubility and an absorption wavelength ranging from 600 to 660 nm [68]. MB is a low-cost, clinically approved compound with well-established antimicrobial activity. Its use in aPDT does not require bioconversion, allowing for a rapid onset of action [60]. Due to these properties, MB has been widely studied for the treatment of dermatophytosis.

Gnat et al. [59] investigated the impact of MB-mediated aPDT on azole-susceptible and -resistant strains of *Trichophyton verrucosum*. The minimum inhibitory concentration (MIC) of MB-aPDT was 2.5 µg/mL for susceptible isolates and 5.0 µg/mL for resistant ones. Moreover, complete inhibition of all isolates was achieved using 5 µg/mL of MB combined with light doses exceeding 40 J/cm^2^. Similarly, Shen et al. [60] reported that MB-aPDT at 32 µg/mL completely inhibited the growth of conidia from *Trichophyton rubrum* and *Trichophyton interdigitale.* Interestingly, no differences in MIC values were observed between terbinafine-susceptible and -resistant strains, suggesting that MB-aPDT acts through strategies that overcome the resistance mechanisms of resistant isolates.

In addition to its effects against conidia, MB-aPDT significantly decreased the viability of *Trichophyton mentagrophytes*, *T. rubrum*, and *Nannizzia gypsea* (formerly *Microsporum gypseum*) biofilms, leading to a reduction in colony-formation unit (CFU) counts exceeding 4 log_10_. Scanning electron microscopy (SEM) images revealed morphological alterations in the biofilms, including hyphal rupture and structural deterioration following treatment [62]. Biofilms are highly structured microbial communities that protect microorganisms from pH fluctuations, nutrient limitations, and harmful external agents, including antimicrobials [69]. Burkharta et al. [70] proposed that biofilms may contribute to treatment resistance and the recurrence of dermatophytosis. However, since in vivo visualization of dermatophyte biofilms has not yet been achieved, further studies are needed to confirm the association between these structures and infection recurrence.

To enhance its antifungal effects, MB-aPDT has also been combined with other compounds. Askari et al. [61] investigated the impact of combined aPDT with both MB and rhamnolipid (MB-Rh-aPDT) on dermatophytic biofilms. MB-Rh-aPDT inhibited biofilm formation by *T. mentagrophytes*, *T. rubrum*, *T. verrucosum*, *Microsporum canis*, and *N. gypsea*. SEM analysis revealed that biofilms exposed to MB-Rh-aPDT during formation exhibited structural damage, including cracks, holes, fractures, twists, and wrinkles in the mycelium, confirming its anti-biofilm effects.

Chen et al. [57] investigated the effects of MB-aPDT, both alone and in combination with itraconazole, fluconazole, terbinafine, and ciclopirox, against biofilms of *T. rubrum*, *T. mentagrophytes*, and *N. gypsea*. Combination therapy of MB-aPDT with itraconazole, fluconazole, and ciclopirox exhibited synergistic effects, significantly reducing biofilm viability and metabolic activity. These outcomes were probably associated with structural alterations observed in the biofilms, including hyphal fragmentation and disorganization of the extracellular matrix. In another study, the combination of MB-aPDT and terbinafine led to clinical improvement in patients with onychomycosis, with the synergistic effects accelerating the healing of severe lesions [58]. Interestingly, the combination therapy yielded promising results with antifungals from different chemical classes and mechanisms of action, reinforcing the potential of MB-aPDT as an adjunctive therapy.

#### 2.1.2. 5-Aminolevulinic Acid

Researchers have analyzed the effects of 5-aminolevulinic acid (ALA) as a PS for treating dermatophytosis, including severe clinical manifestations such as kerion and Majocchi’s granuloma. ALA, an endogenous non-proteinogenic amino acid, serves as a precursor to protoporphyrin IX (PpIX). Upon cellular uptake, ALA enhances the production and accumulation of PpIX. When exposed to red light (620 to 750 nm) in the presence of molecular oxygen, the accumulated PpIX generates ROS, ultimately leading to cell death [71,72].

ALA-aPDT has been investigated as an alternative treatment for a refractory case of Majocchi’s granuloma [63], a granulomatous and inflammatory dermatophytic infection that can manifest as pustules, nodules, infiltrative plaques, or even deep abscesses and subcutaneous induration [73]. Shi et al. [63] reported that ALA-aPDT successfully eliminated Majocchi’s granuloma lesions, as confirmed by mycological and pathological examinations. Subsequent in vitro analyses showed that ALA-PDT inhibited the proliferation of *Trichophyton tonsurans*, while transmission electron microscopy (TEM) revealed significant cellular damage, including a blurred cell wall, condensed chromatin, and a swollen endoplasmic reticulum. In addition, in vivo assays demonstrated a marked improvement in skin lesions after treatment with ALA-aPDT.

Recently, Ji et al. [64] evaluated the effects of combining ALA-aPDT and terbinafine in patients with kerion and facial ulcers secondary to *T. rubrum* infection. A significant reduction in pustules and purulent discharge was observed after the first session of ALA-aPDT, with complete lesion healing achieved after three sessions. Pain and edema were managed with analgesia and local cooling, and no severe adverse effects were reported. In another study, Zhang et al. [32] investigated a combination of ALA-aPDT with itraconazole, terbinafine, and butenafine for treating *Microsporum canis*-induced *tinea capitis*. All cases demonstrated clinical improvement, with complete lesion resolution and no mycological growth detected during follow-up. While erythema and pruritus were reported, no serious adverse effects occurred. Importantly, hair regrowth was observed in previously affected areas following treatment completion.

#### 2.1.3. Hypericin

Hypericin (Hyp), a chemically synthesized polycyclic aromatic naphthodianthrone, has been studied as a mediator of aPDT for antifungal applications. This lipophilic compound, when formulated in clinical solvents, becomes highly soluble in physiological systems, demonstrating good permeability, promising pharmacokinetics, and photodynamic properties [74].

Conrado et al. [65] investigated the effects of Hyp-aPDT for treating onychomycosis caused by *T. rubrum*. The therapy effectively reduced fungal growth, particularly by inhibiting the germination of microconidia. Furthermore, mycological cure was achieved in patients after four Hyp-aPDT sessions, with no reports of pain, burning sensation, or adverse effects. In another study, aPDT mediated by nanoencapsulated Hyp in micelles significantly reduced the viability of *T. rubrum* conidia, exhibiting fungicidal activity at concentrations ranging from 3.12 to 50 mM. Moreover, the therapy decreased both the metabolic activity and viability of biofilms, as confirmed by SEM images showing a reduction in total biomass, damage to the extracellular matrix, and alterations in the fungal cell wall. Hyp-aPDT also impaired cell adhesion to surfaces, thereby preventing biofilm formation and inhibiting the development of early biofilm layers [66].

#### 2.1.4. Other Photosensitizers

In addition to the well-studied PSs—MB, ALA, and Hyp—the effects of other products, such as aloe-emodin, 2-hydroxychalcone, and tetra-cationic porphyrins with peripheral platinum (II) and palladium (II) complexes, have also been investigated against dermatophytes.

Ma et al. [67] investigated the effects of aloe-emodin (AE)-mediated aPDT against *T. rubrum*. AE is a natural anthraquinone extracted from traditional Chinese medicinal herbs, known for its broad biological properties and, mainly, its structural similarity to Hyp [75]. In their study, Ma et al. [67] observed that the cellular uptake of AE occurred within 2 h, leading to an increased production of ROS, which ultimately resulted in cell wall damage and a reduction in cell membrane thickness. Furthermore, AE-aPDT completely inhibited fungal growth in *T. rubrum*-caused *tinea corporis* in a guinea pig model and *tinea unguium* in an ex vivo model.

Bila et al. [23] compared the activity of free 2-hydroxychalcone (Hyd) and its role as a PS against *T. rubrum* and *T. mentagrophytes*. Chalcones are polyhydroxylated compounds widely found in vegetables, fruits, and edible plants [76]. The MIC values of Hyd-aPDT ranged from 2 to 7.8 µg/mL. Hyd specifically targeted ergosterol in the fungal cell membrane, increasing ROS production and inducing cell death through apoptosis and necrosis pathways. Hyd-aPDT also demonstrated promising antibiofilm activity against both early-stage and mature biofilms. Moreover, Hyd and Hyd-aPDT were biocompatible with keratinocytes, supporting the potential use of Hyd-aPDT as an alternative treatment for *Trichophyton* spp. infections.

Pinto et al. [47] investigated the effects of tetra-cationic porphyrins with peripheral platinum (II) and palladium (II) complexes (3PtP) as PSs for aPDT in combination with itraconazole against dermatophytes. Broad-spectrum fungicidal activity on *T. rubrum*, *T. tonsurans*, *T. mentagrophytes*, *M. canis*, *N. gypsea*, *Microsporum nanum*, and *Epidermophyton floccosum* was shown by 3PtP-aPDT at concentrations ranging from 0.62 to 5 µM. The combination of 3PtP-aPDT and itraconazole exhibited a synergistic effect, enhancing its antidermatophytic activity. As expected, 3PtP-aPDT generated ROS, such as singlet oxygen, hydroxyl radicals, and superoxide, which contributed to fungal inactivation.

### 2.2. aPDT for the Treatment of Superficial and Cutaneous Infections Caused by Candida spp.

*Candida* spp. are the primary fungal pathogens associated with human infections, commonly found as commensals on the skin, gastrointestinal tract, genitourinary tract, and oral cavity of healthy individuals. The annual incidence of *Candida* infections is estimated at 4 million cases worldwide [2]. Among these, oral candidiasis represents one of the most prevalent clinical manifestations, accounting for approximately 2 million cases per year worldwide [77]. In addition, *Candida* spp. infections also affect the genitourinary tract of approximately 75% of women at least once in their lifetime. Of these, 40–45% will experience recurrent episodes, and approximately 10–20% will develop complicated forms of the infection [78,79].

In this context, *Candida albicans* remains the most frequently isolated fungal pathogen in humans. However, a notable rise in infections caused by non-*albicans Candida* species, including *Candida tropicalis*, *Candida parapsilosis*, *Pichia kudriavzevii* (formerly *Candida krusei*), and *Nakaseomyces glabratus* (formerly *Candida glabrata*), has been observed in recent years [80,81]. These species exhibit high adaptability to various host environments and can cause a broad spectrum of clinical manifestations—from superficial mucocutaneous infections to invasive bloodstream infections [82,83].

The conventional treatment of *Candida* infections relies primarily on three major classes of antifungal agents: polyenes, azoles, and echinocandins [84]. However, these therapies are often associated with several limitations and the emergence of antifungal-resistant *Candida* strains, such as multidrug-resistant *Candidozyma auris* (formerly *Candida auris*), an emerging global pathogen linked to high mortality rates and nosocomial outbreaks [22,85]. Given these concerns, aPDT has gained attention as a promising alternative or adjunctive treatment for candidiasis. In this context, various PSs, including MB, toluidine blue, ALA, photodithazine, and a range of newly identified compounds, are under investigation (Table 2).

#### 2.2.1. MB

As observed for dermatophytes, MB is one of the most extensively studied PSs for aPDT in the treatment of *Candida* spp. infections, particularly those affecting the oral cavity. In a clinical context, Al-Aali et al. [87] evaluated the impact of MB-aPDT on *Candida* growth and oral health-related quality of life in patients with denture stomatitis. Denture stomatitis is a highly prevalent condition among denture wearers and is frequently associated with infection by *Candida* spp. [99]. Treatment with MB-aPDT led to a significant reduction in *Candida* counts after 14 days, alongside a modest improvement in patients’ quality of life. The combined use of MB-aPDT and miconazole enhanced antifungal efficacy and resulted in greater improvement in quality-of-life scores, suggesting that this combination therapy could serve as a promising alternative treatment for managing denture stomatitis.

Promisingly, MB-aPDT also demonstrated efficacy against the multidrug-resistant pathogen *C. auris* [86], a species recognized for its resistance to major antifungal classes, largely due to the overexpression of efflux pumps [100]. MB-aPDT significantly disrupted *C. auris* biofilms, with inhibition rates exceeding 99% following 300 s of treatment, even in fluconazole-resistant strains. Gene expression analysis revealed an upregulation of efflux pump genes *CDR1* and *MDR1* after MB-aPDT, while *CDR2* expression remained unaffected. Interestingly, this upregulation did not compromise the effectiveness of MB-aPDT, suggesting that efflux pump activity alone may not confer resistance to photodynamic therapies.

To enhance the effects of MB, Soares et al. [88] developed MB-loaded polymeric micelles for use in aPDT targeting *C. albicans* biofilms. The micelles, composed of polymeric nanosystems, can improve treatment outcomes by facilitating the penetration of PSs into the biofilm matrix and controlling their release [101]. The aPDT using MB-loaded micelles significantly reduced yeast viability within 5 min, achieving complete fungal elimination after 30 min of PIT. However, differences in efficacy between free MB and MB-loaded micelles were observed only at 15 and 30 min of PIT, emphasizing the importance of optimizing treatment parameters for clinical applications of aPDT.

#### 2.2.2. Toluidine Blue

Toluidine blue O (TBO) is a basic thiazine metachromatic dye partially soluble in both water and alcohol. It possesses several characteristics favorable for clinical applications, including low cost, minimal cytotoxicity to host cells, low excitation energy, high singlet oxygen quantum yield, and strong affinity for cellular components and target cell membranes [102,103]. These features make TBO a widely used PS in aPDT.

Afrasiabi et al. [90] investigated the susceptibility of *C. albicans* mature biofilms formed on dental implants to TBO-aPDT, both alone and in combination with hydrogen peroxide (HP–TBO-aPDT). While both treatment modalities reduced the fungal burden, the combined therapy exhibited significantly greater antifungal efficacy. Importantly, the HP–TBO-aPDT treatment resulted in a 2.47-fold increase in ROS production, indicating that the synergistic effect between TBO-aPDT and HP can enhance the antifungal activity on *C. albicans* biofilms adhered to implant surfaces.

TBO-aPDT also demonstrated effects on dual-species biofilms composed of *C. albicans* and *P. kudriavzevii* [89]. The findings of the related study revealed a reduction in metabolic activity between 22% and 33% following treatment. Interestingly, *C. albicans* showed a notable decrease in viable counts, whereas *P. kudriavzevii* remained largely unaffected, highlighting differential susceptibility profiles between these two related species. This study underscores the importance of evaluating antifungal effects on complex biofilm models, which better mimic the real-life conditions found in various anatomical sites and on medical devices.

#### 2.2.3. ALA

ALA has also shown promising potential as a PS in aPDT for combating *Candida* spp. infections, with several studies highlighting its antifungal efficacy. Shi et al. [91] evaluated the inhibitory effects of ALA-PDT against *C. albicans* biofilms and observed ultrastructural alterations consistent with sublethal cellular damage. TEM images revealed notable changes, including cytoplasm degradation, nuclear condensation, and mitochondrial swelling. At the molecular level, ALA-PDT significantly induced apoptosis (19.4%), markedly higher than in the control groups or those treated with ALA or light alone. Moreover, gene expression analysis showed the downregulation of *HWP1*, *ALS3*, *UME6*, and *HGC1*, genes that are critical for biofilm formation and maintenance.

Clinically, ALA-aPDT also demonstrated effectiveness in treating cutaneous *Candida* spp. infections. In a case reported by He and Lu [92], a patient with *C. tropicalis*-associated ulcerative lesion, unresponsive to conventional therapies, showed significant improvement following a combined regimen of surgical debridement, oral itraconazole, and ALA-aPDT. Clinical remission was achieved after a single aPDT session, with no recurrence observed after two months of follow-up. Similarly, Wang et al. [93] successfully employed ALA-aPDT to treat a *C. albicans*-induced cutaneous granuloma, resulting in complete healing within two weeks post-treatment. Altogether, these findings support ALA-aPDT as a promising alternative for difficult-to-treat *Candida* infections, offering localized and effective treatment with good clinical outcomes.

#### 2.2.4. Photodithazine

Photodithazine (PDZ), a chlorine e6 derivative extracted from *Spirulina platensis* [104], has also demonstrated efficacy as a PS against *Candida* spp. infections in vitro and in vivo. As a second-generation, water-soluble PS, PDZ offers several advantages, including high stability during storage and strong absorption in the red region of the light spectrum (around 660 nm), which enhances tissue penetration [105].

Dias et al. [94] investigated the antifungal effects of successive applications of PDZ-aPDT, both as monotherapy and in combination with fluconazole, against *C. albicans*. The study demonstrated complete inactivation of *C. albicans* in planktonic cultures and biofilms after three and five treatment sessions, respectively, highlighting the well-documented increased resistance of biofilms to antifungal strategies. PDZ-aPDT alone achieved a reduction of 6.3 log_10_ CFU/mL in planktonic cultures and 6.1 log_10_ CFU/mL in biofilms. When combined with fluconazole, these values were 7 log_10_ CFU/mL and 6.7 log_10_ CFU/mL, respectively, indicating a synergistic enhancement of antifungal activity. The observed antibiofilm effects may be partly attributed to the downregulation of *ALS1* and *HWP1* genes following PDZ-aPDT, as reported by Jordão et al. [95]. These genes encode molecules involved in the initial stages of biofilm development, hyphal formation, and tissue invasion [106,107]. Furthermore, PDZ-aPDT reduced the expression of genes associated with oxidative stress defense, specifically the transcription factor *CAP1*, as well as *CAT1* (catalase) and *SOD1* (superoxide dismutase). Altogether, these results suggest that PDZ-aPDT not only promotes ROS generation but also impairs fungal antioxidant defense mechanisms, thereby contributing to its potent antifungal action.

#### 2.2.5. Other PSs

In addition to MB, TBO, ALA, and PDZ, several other PSs, such as AE and indocyanine green, have been evaluated for their anti-*Candida* effects in aPDT. Ma et al. [96] assessed the efficacy of AE-aPDT on *C. albicans* strains and reported no significant dark toxicity of AE. Confocal laser scanning microscopy revealed substantial structural damage to the fungal cell wall, cytoplasm, and nucleus following AE-aPDT. In addition, AE-aPDT achieved a 6.5 log_10_ CFU/mL reduction in both azole-sensitive and -resistant strains, highlighting its potential as a broad-spectrum antifungal strategy. Similarly, Mardani and Kamrani [97] investigated the effectiveness of aPDT mediated by indocyanine green (ICG), a water-soluble anionic tricarbocyanine dye, on fluconazole-sensitive and -resistant *C. albicans*. Promisingly, ICG-aPDT not only demonstrated antifungal activity against planktonic cells but also effectively inhibited biofilm formation in both strains.

Tetra-cationic porphyrins containing peripheral bipyridyl Pt(II) complexes and riboflavin have also been explored as mediators in aPDT for the treatment of oral candidiasis. Garcia et al. [48] evaluated the antifungal efficacy of functionalized cationic porphyrin as a PS in aPDT (3-Pt-aPDT) against denture stomatitis and burn wounds caused by *C. albicans*. The viability of planktonic cells was completely inhibited by 3-Pt-aPDT in 40 s, also extending to biofilms, in which the reduction was 4 log_10_ CFU. The therapy inhibited the filamentation of yeasts and increased ROS production, leading to cell wall damage, as demonstrated by TEM images. Importantly, 3-Pt-aPDT showed antifungal efficacy against denture stomatitis biofilms in a microcosm model and burn wounds in *Galleria mellonella*, indicating its potential for treating *C. albicans*-associated infections.

In another study, Alshehri [98] analyzed the effects of riboflavin-mediated aPDT (RF-aPDT) against *C. albicans* biofilms formed on polymethyl methacrylate, a material commonly used in dental prosthetics and prone to *Candida* colonization [108]. RF-aPDT decreased *C. albicans* viability, with superior results compared to the group treated with nystatin. Importantly, RF-aPDT did not compromise the surface integrity of resin, suggesting its suitability as a safe and effective therapeutic approach for managing *Candida*-associated prosthetic infections.

### 2.3. aPDT for the Treatment of Infections Caused by Superficial and Cutaneous Agents Other than Dermatophytes and Candida spp.

Although superficial and cutaneous mycoses are primarily caused by dermatophytes and *Candida* spp., several other fungal species can colonize these anatomical sites and, although less prevalent, may also lead to infections [3,109]. Importantly, some of these etiological agents are capable of invading subcutaneous tissues, resulting in diverse clinical manifestations and difficult-to-treat mycoses [110,111]. In this context, aPDT mediated by various PSs has been proposed as an alternative—and, in some cases, adjunctive—therapeutic approach (Table 3).

#### 2.3.1. ALA

Due to its properties, ALA-aPDT has been studied as a non-invasive and non-toxic approach for treating wound infections caused by yeasts and filamentous fungi, including *Trichosporon asahii*, *Fonsecaea pedrosoi*, *Exophiala spinifera*, and *Curvularia lunata*.

Chen et al. [7] and Lan et al. [113] used ALA-aPDT in combination with azoles to treat trichosporonosis, a superficial mycosis primarily caused by the yeast-like fungus *T. asahii* [7,113]. In both cases, adjunctive treatment with itraconazole and voriconazole led to mycological cure, with negative cultures reported after one month of therapy. In vitro studies further demonstrated that ALA-aPDT reduced the viability of *T. asahii* planktonic cells, including an itraconazole-resistant strain. Moreover, the combination of ALA-aPDT and itraconazole significantly decreased the metabolic activity and biomass of *T. asahii* biofilms, confirming the antifungal effects both on planktonic cells and biofilms.

Another study reported the successful use of ALA-aPDT in the treatment of phaeohyphomycosis caused by *E. spinifera* [112]. Phaeohyphomycosis includes cutaneous, subcutaneous, and systemic infections characterized by the presence of melanized yeast-like cells and/or hyphal elements without the formation of sclerotic cells. In this case, ALA-aPDT was combined with itraconazole and terbinafine. The therapy was well tolerated, with no significant adverse events aside from mild burning and temporary pain. Following treatment, the patient showed marked clinical improvement, with resolution of papules and nodules and negative mycological cultures from skin lesions.

In a separate case, Wang et al. [16] demonstrated the efficacy of ALA-aPDT as a monotherapy for a cutaneous fungal infection caused by *C. lunata*. The treatment resulted in complete resolution of lesions without the need for systemic antifungal agents. ALA-aPDT was well tolerated, with no discomfort or serious adverse effects and no recurrence observed after a six-month follow-up. Supporting in vitro experiments further confirmed that ALA at concentrations above 2.5% significantly inhibited the growth of *C. lunata* during aPDT, whereas ALA or light exposure alone had no antifungal effect.

#### 2.3.2. MB

Two recent studies have explored the use of MB in aPDT to treat infections caused by *Sporothrix globosa* and *Malassezia* spp. Li et al. [114] evaluated the fungicidal effects of MB-aPDT against *S. globosa*, the etiological agent of sporotrichosis—a subcutaneous mycosis with cutaneous and lymphocutaneous manifestations. MB-aPDT significantly reduced the viability of *S. globosa* strains, and SEM images revealed notable morphological alterations in conidia, including shrinkage and elongation. In addition, the combination of MB-aPDT with itraconazole enhanced the antifungal activity in vitro, with the ability to reduce lesions in a murine model of sporotrichosis.

The clinical efficacy of MB-aPDT was also investigated in patients with pityriasis versicolor [30], a superficial fungal infection caused by *Malassezia* spp.—a lipophilic yeast that naturally colonizes the skin [116]. All patients achieved clinical and mycological cure after two MB-aPDT sessions, with no reports of adverse effects, recurrence, or discomfort. The therapy also yielded favorable cosmetic outcomes, highlighting MB-aPDT as a safe and effective approach for managing pityriasis versicolor.

#### 2.3.3. Other PSs

Strategies using haematoporphyrin monomethyl ether (HMME), AE, and methyl aminolevulinate as PSs in aPDT have been investigated for their antifungal activity against *Malassezia furfur*, another major causative agent of pityriasis versicolor. HMME is a second-generation porphyrin PS characterized by its high selectivity, low toxicity, and high yield of singlet oxygen [117]. In this context, Cui et al. [8] observed that both HMME-aPDT and AE-aPDT reduced the viability of *M. furfur* clinical isolates, with AE-aPDT showing superior antifungal efficacy. Both therapies led to disruption of the fungal cell wall, associated with elevated intracellular ROS levels and inhibition of secreted protease and lipase activities—enzymes linked to fungal virulence.

In another study, Arriba et al. [24] investigated the effects of water-filtered infrared A (wIRA) alone and in combination with methyl aminolevulinate (MAL-wIRA) against *M. furfur*, both in monoculture and co-cultured with keratinocytes and dendritic cells. wIRA is an experimental light-based therapy that enhances tissue oxygenation and promotes wound healing through cell metabolism modulation [118]. MAL, a prodrug converted into the active PS PpIX, augmented the antifungal effects when combined with wIRA. Both wIRA alone and MAL-wIRA treatments reduced fungal counts, with MAL-wIRA showing enhanced efficacy. Importantly, both therapies exhibited good biocompatibility with keratinocytes but were not considered safe for dendritic cells. In co-cultures of *M. furfur* with keratinocytes, both treatments decreased fungal burden and suppressed the expression of the proinflammatory cytokine IL-6, suggesting a potential immunomodulatory role and a mechanism aligned with wound healing pathways [119].

To enhance the antifungal activity of PSs, Kodedová et al. [115] developed a nanoformulation of 5,10,15,20-tetraphenylporphyrin (TPP) using sulfonated polystyrene nanoparticles (TPP-NPs). The antifungal effects of this formulation were evaluated on *Hortaea werneckii*, the etiological agent of tinea nigra—a superficial mycosis characterized by hyperpigmented macules on the stratum corneum of the palms and soles [6]. *H. werneckii* showed susceptibility to TPP-NPs-mediated aPDT, with increased singlet oxygen production being a major contributing factor to fungal inactivation. Although the cell wall of *H. werneckii* contains melanins—particularly 1,8-dihydroxynaphthalene (DHN)-melanin—which are typically associated with protection against oxidative stress, the aPDT treatment significantly reduced cell viability. These findings suggest that DHN-melanin alone may not be sufficient to counteract the oxidative damage induced by the singlet oxygen species generated during TPP-NPs-aPDT.

### 2.4. Limitations and Future Directions for aPDT

**Figure 2:**
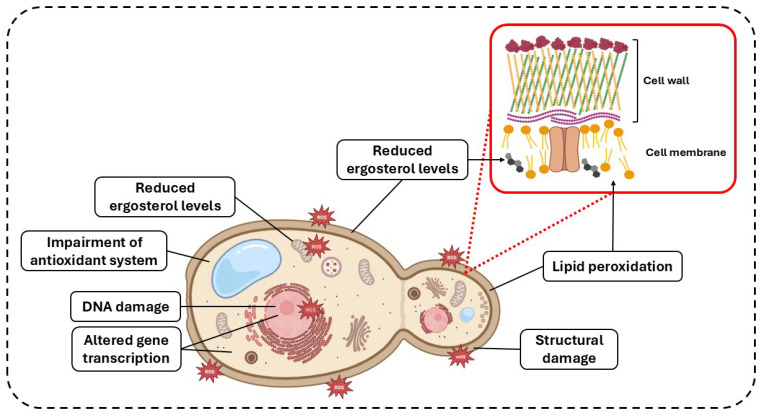
**Summary of the mechanisms of action of antimicrobial photodynamic therapy (aPDT) on fungal cells.** aPDT acts on fungal cells by increasing the production of reactive oxygen species (ROS). When these ROS interact with the cell membrane and mitochondria, they can induce lipid peroxidation and reduce ergosterol content. In addition, ROS can lead to DNA damage, alter gene expression, impair the functionality of fungal antioxidant enzymes, and ultimately cause structural damage. Image created using Biorender (www.biorender.com, accessed on 27 May 2025).

Several studies mentioned in this review have demonstrated the efficacy of aPDT against fungal cells through multiple mechanisms of action, which are shared across yeast, filamentous fungi, and biofilms. The increased production of ROS induced by aPDT leads to cellular damage, including lipid peroxidation, reduced ergosterol levels, DNA damage, alterations in gene transcription, and impairment of fungal antioxidant enzymes (Figure 2). Collectively, these effects result in the partial or complete inhibition of fungal viability.

Despite these promising in vitro and in vivo outcomes, aPDT presents limitations that must be addressed to facilitate its clinical application. These limitations are primarily related to the physicochemical properties of the PS and to certain aspects of the aPDT protocol. One of the main challenges associated with PSs is their hydrophobicity, which impairs their solubility and bioavailability in biological systems, potentially limiting tissue penetration and efficacy [43]. In this review, the PSs with the most well-documented antifungal activity in vitro and in vivo are ALA and MB. However, ALA requires pre-treatment and a longer PIT since it must undergo enzymatic conversion into PpIX for effectiveness [71,72]. In skin and mucosal infections, MB does not require pre-treatment and has demonstrated superior results compared to ALA. Nonetheless, both photosensitizers (PSs) have limitations in treating onychomycosis, particularly in cases of deep nail hyperkeratosis, where micro-abrasion of the nail plate is necessary to enhance PS uptake. When comparing penetration into the nail plate, MB exhibits superior absorption compared to ALA, which translates to higher rates of complete healing [120,121]. In addition, many PSs lack selectivity for fungal cells, increasing the risk of off-target effects and damage to host tissues. Other limitations include the encapsulation process, which can be complex, and the overall cost of certain PS compounds [26,45,122].

From a procedural perspective, the main limitations are associated with the lack of standardized protocols, particularly regarding the selection of the ideal PS and irradiation parameters. In addition, prolonged PIT and extended irradiation periods pose challenges for clinical application. For instance, treatment regimens requiring PITs of up to 4 h and irradiation times of 30 min may not be practical for clinical use [16,63,112]. Shorter PITs are advantageous in clinical applications, as they can reduce clinical treatment duration without compromising photodynamic antifungal efficacy [123]. In addition, aesthetic concerns may hinder patient adherence to therapy, as some PSs, such as MB and TBO, can temporarily stain mucosal tissues, which could be perceived as undesirable in cosmetic settings [124,125].

To overcome these challenges, strategies focusing on optimizing the physicochemical properties of PSs through advanced drug delivery systems have been evaluated. Nanostructured carriers, including nanoparticles, micelles, and nanotubes, can enhance PS solubility, stability, and selectivity [66,88,115]. Furthermore, engineering approaches to modify cell surfaces and the use of adjunctive treatments to improve PS uptake may significantly enhance the antifungal efficacy of aPDT [45]. Moreover, the development of PSs designed for oxygen-independent photodynamic strategies may broaden the spectrum of fungal infections treatable by aPDT. This approach enables the generation of reactive species even in anaerobic or hypoxic environments—conditions commonly found in deep-seated or poorly vascularized infections [41,42,43]. However, further studies are needed to fully elucidate the mechanisms and therapeutic potential of this strategy.

In this context, the development of novel PSs, their nanoformulation, and the application of techniques to improve their physicochemical properties, along with the advancement of PSs designed for oxygen-independent activity, are expected to expand the spectrum of infections treatable by aPDT and support its successful clinical translation as an effective antifungal therapy.

## 3. Conclusions

Several in vitro and in vivo studies have shown the antifungal efficacy of aPDT in treating superficial, skin, and mucosal mycoses. PSs such as MB and ALA have demonstrated broad-spectrum antifungal activity against dermatophytes, *Candida* spp., and other pathogenic fungi. Promisingly, aPDT has proven effective against both susceptible and resistant strains, highlighting its potential to combat multidrug-resistant isolates and even reduce the emergence of antifungal resistance. In addition, several studies have reported successful management of fungal infections unresponsive to conventional antifungal therapies using aPDT. Importantly, these cases were associated with favorable clinical outcomes, including mycological cure, absence of adverse effects, low recurrence rates, and overall treatment safety. While the antifungal activity of well-established PSs is supported by robust in vitro and in vivo data, newly developed PSs still require further investigation to clarify their physicochemical properties, as well as their pharmacokinetic and pharmacodynamic profiles.

Although this review highlights the effects of aPDT for the treatment of superficial, skin, and mucosal fungal infections, its clinical application faces several limitations. These include the penetration of PSs into anatomical sites such as nails, hydrosolubility, and the need for pre-treatment, as observed with ALA. In addition, prolonged PIT and extended irradiation periods may reduce patient adherence and ultimately limit its clinical use. However, materials engineering approaches focused on optimizing the physicochemical properties of PSs through nanostructuring and surface treatments offer promising solutions to overcome these challenges and enhance the clinical applicability of aPDT.

On the other hand, aPDT presents significant advantages, including non-invasiveness, minimal to no side effects, and a low probability of resistance development. Furthermore, its ability to be combined with other therapeutic strategies, along with the low cost of instrumentation, operational simplicity, and rapid results, makes aPDT a cost-effective and accessible alternative, especially in comparison to antifungal drugs, which often require months of continuous use.

In summary, although aPDT has limitations, current evidence supports the potential of aPDT as a valuable strategy for managing cutaneous, mucosal, and superficial fungal infections and for addressing the growing challenge of antifungal resistance.

## Figures and Tables

**Table 1:** Summary of antimicrobial photodynamic therapy (aPDT) parameters used against dermatophytes, including targeted species and key outcomes.

Reference	Species	Photosensitizer	Irradiation Parameters	Main Results
Chen et al. [57]	*Trichophyton rubrum*, *Trichophyton mentagrophytes*, and *Nannizzia gypsea*	Methylene blue (MB)	Pre-irradiation time (PIT): 2 h 635 nm, 100 mW/cm^2^, 60 J/cm^2^, 10 min	Antifungal activity against conidia and biofilms Structural disruption of biofilms
Alberdi and Gómez [58]	Not specified	MB	PIT: 3 min 635 nm, 62 mW/cm^2^, 37 J/cm^2^, 10 min	Significant clinical improvement Synergistic effects with terbinafine Well-tolerated treatment
Gnat et al. [59]	*Trichophyton verrucosum*	MB	PIT: 10 min 635 nm, 58 mW/cm^2^, 17 to 1750 s, 1–100 J/cm^2^	Antifungal effects on both sensitive and resistant strains Antibiofilm effects
Shen et al. [60]	*T. rubrum* and *Trichophyton interdigitale*	MB	PIT: 3 min, 30 min and 3 h 635 nm, 37 J/cm^2^, 485 s	Inhibition of conidia growth Antifungal effects on both terbinafine-sensitive and -resistant strains
Askari et al. [61]	*T. mentagrophytes*, *T. rubrum*, *T. verrucosum*, *Microsporum canis*, and *N. gypsea*	MB	PIT: 3 h 600 and 635 nm, 100 MW/cm^2^, 10 min	Inhibitory effects on biofilms Structural damage to biofilms Synergism with rhamnolipid
Chen et al. [62]	*T. rubrum*, *T. mentagrophytes*, and *N. gypsea*	MB	PIT: 3 h 635 ± 10 nm, 100 mW/cm^2^, 60 J/cm^2^, 10 min	Antibiofilm effects Hyphal disruption and structural damage in biofilms
Shi et al. [63]	*Trichophyton tonsurans*	5-aminolevulinic acid (ALA)	PIT: 5 h 633 nm, 40 mW/cm^2^, 200 J/cm^2^	Antifungal activity Improvement of ulcerative lesions Mycological cure
Zhang et al. [32]	*M. canis*	ALA	PIT: 3 h 630–635 nm, 200–300 mW/cm^2^, 60–80 J/cm^2^, 10 to 25 min	Fungal eradication Lesion improvement and regrowth of hair No recurrence Synergistic activity with antifungals
Ji et al. [64]	*T. rubrum*	ALA	PIT: 3 h 635 nm, 60 and 90 mW/cm^2^, 72 and 108 J/cm^2^	Complete lesion healing Effective in recurrent infections Mild adverse effects
Conrado et al. [65]	*T. rubrum*	Hypericin (Hyp)	PIT: 30 m 400–800 nm, 30 and 67 mW/cm^2^, 37.8 and 66 J/cm^2^	Antifungal activity Mycological cure No adverse effects
Fernandes et al. [66]	*T. rubrum*	Hyp	PIT: 2 h 596–600 nm, 37.9 J/cm^2^	Antifungal activity Antibiofilm effects Inhibition of cell adhesion
Ma et al. [67]	*T. rubrum*	Aloe-emodin	PIT: 1, 2 and 3 h 435 ± 10 nm, 40 mW/cm^2^, 2.4–72 J/cm^2^	Antifungal activity on both conidia and hyphae Effective in treating both *tinea corporis* and *tinea unguium* Healing and hair regrowth post-treatment
Bila et al. [23]	*T. rubrum* and *T. mentagrophytes*	2-hydroxychalcone	PIT: 10 min 455–492 nm, 58 mW/cm^2^, 150 J/cm^2^	Antifungal activity Activity against mature biofilms Biocompatibility with mammalian cells
Pinto et al. [47]	*T. rubrum*, *T. tonsurans*, *T. mentagrophytes*, *M. canis*, *N. gypsea*, *M. nanum*, and *E. floccosum*	Tetra-cationic porphyrins with peripheral platinum (II) and palladium (II) complexes	400–800 nm, 25 mW/cm^2^, 180 J/cm^2^, 120 min	Antifungal activity Increased ROS generation Synergism with itraconazole

**Table 2:** Summary of antimicrobial photodynamic therapy (aPDT) parameters used against *Candida* spp. and related species, including targeted species and key outcomes.

Reference	Species	Photosensitizer	Irradiation Parameters	Main Results
Štefánek et al. [86]	*Candidozyma auris*	Methylene blue (MB)	Pre-irradiation time (PIT): 1 h 660 nm, 190 mW/cm^2^, 15, 23, and 58 J/cm^2^, 79, 120, and 300 s	Upregulation of efflux pumps genes (*CDR1* and *MDR1*) Antibiofilm effects
Al-Aali et al. [87]	*Candida* spp.	MB	PIT: 5 min 600 nm, 100 mW, 3527 mW/cm^2^, 9 J	Antifungal activity Synergism with miconazole Resolution of palatal inflammation
Soares et al. [88]	*Candida albicans*	MB	PIT: 5, 15 and 30 min 660 nm, 19 mW/cm^2^, 15 J/cm^2^, 13 min and 16 s	Decreased fungal viability Inhibition of biofilm formation
Rodrigues et al. [89]	*C. albicans* and *P. kudriavzevii*	Toluidine Blue O (TBO)	PIT: 10 min 630 nm, 0.069 W, 30 J/cm^2^, 165 s	Decreased viability of dual-species biofilms
Afrasiabi et al. [90]	*C. albicans*	TBO	635 and 980 nm, 200 and 800 mW, 6 and 24 J/cm^2^, 60 s	Reduction of fungal viability Increased reactive oxygen species (ROS) levels
Shi et al. [91]	*C. albicans*	5-aminolevulinic acid (ALA)	PIT: 5 h 635 nm, 100 mW/cm^2^, 300 J/cm^2^, 50 min	Structural damage to biofilms Induced cell apoptosis Downregulation of biofilm-associated genes
He and Lu [92]	*C. tropicalis*	ALA	PIT: 4 h 635 nm, 177 mW/cm^2^, 120 J/cm^2^, 15 min	Synergism with surgical debridement and itraconazole Good clinical outcomes No significant adverse events No recurrence
Wang et al. [93]	*C. albicans*	ALA	PIT: 2 h 633 ± 10 nm, 80 mW/cm^2^, 25 min	Clinical improvement No significant adverse events No recurrence
Dias et al. [94]	*C. albicans*	Photodithazine (PDZ)	PIT: 20 min 660 nm, 34 mW/cm^2^, 18 J/cm^2^, 9 min	Activity on planktonic cells and biofilms Synergism with fluconazole
Jordão et al. [95]	*C. albicans*	PDZ and curcumin	PIT: 20 min PDZ: 660 nm, 71.7 mV/cm^2^, 37.5 and 50 J/cm^2^, 9 and 12 min CUR: 450 nm, 30 mV/cm^2^, 37.5 and 50 J/cm^2^, 21 and 27 min	Downregulation of biofilm-associated genes Downregulation of genes associated with oxidative stress defense
Ma et al. [96]	*C. albicans*	Aloe emodin	PIT: 30 min 400–780 nm, 80 mW/cm^2^, 2.4, 4.8, 14.4, and 24 J/cm^2^, 30, 60, 180, and 300 s PIT: 0, 10, 30 and 60 min 400–780 nm, 80 mW/cm^2^, 4.8 J/cm^2^, 1 min	Inhibitory effects on planktonic cells Structural damage of fungal cells
Mardani and Kamrani [97]	*C. albicans*	Indocyanine green	PIT: 30 min 810 nm, 300 mW, 228 J/cm^2^, 2 min	Antifungal activity Decreased biofilm formation
Alshehri et al. [98]	*C. albicans*	Riboflavin	PIT: 10 min 450 nm, 25 mW/cm^2^, 15 J/cm^2^, 10 min	Antifungal activity No deterioration of acrylic denture material
Garcia et al. [48]	*C. albicans*	Tetra-cationic porphyrins with peripheral platinum (II) and palladium (II) complexes	PIT: 5 to 80 s 420 nm, 1250 mW, 0.16 W/cm^2^, 0.79–12.78 J/cm^2^	Activity against planktonic cells and biofilms Reduced fungal filamentation Increased in vivo survival in a burn wound model

**Table 3:** Summary of antimicrobial photodynamic therapy (aPDT) parameters used against superficial and cutaneous agents other than dermatophytes and *Candida* spp., including targeted species and key outcomes.

Reference	Species	Photosensitizer	Irradiation Parameters	Main Results
Liu et al. [112]	*Exophiala spinifera*	5-aminolevulinic acid (ALA)	Pre-irradiation time (PIT): 4 h 633 nm, 120 mW/cm^2^, 25 min	Clinical improvement Resolution of papules and nodules Mycological cure No significant adverse events
Lan et al. [113]	*Trichosporon asahii*	ALA	PIT: 1 h 635 ± 10 nm, 60 mW/cm^2^, 36, 72, and 108 J/cm^2^ 4 h (in vivo)	Reduced planktonic cells Decrease adherence and biofilm formation in vitro Wound healing Mycological cure
Wang et al. [16]	*Curvularia lunata*	ALA	PIT: 4 h 635 nm, 80 mW/cm^2^, 30 min	Complete resolution of lesions No discomfort or serious adverse effects No recurrence
Chen et al. [7]	*Trichosporon asahii*	ALA	4 h	Improvement of ulcerative lesions
Li et al. [114]	*Sporothrix globosa*	Methylene blue (MB)	PIT: 30 min 640 nm, 40 J/cm^2^, 30 min	Antifungal activity against conidia Synergism with itraconazole in vitro Reduction of lesions No systemic dissemination
Alberdi and Gomez [30]	*Malassezia* spp.	MB	PIT: 3 min 630 ± 5 nm, 37 J/cm^2^, 50 min	Complete cure No recurrence and adverse effects Good cosmetic outcome
Cui et al. [8]	*Malassezia furfur*	Haematoporphyrin monomethyl ether Aloe emodin	PIT: 30 min 400–780 nm, 40 mV/cm^2^, 72 J/cm^2^ (15 min) and 96 J/cm^2^ (20 min)	Antifungal activity Increased reactive oxygen species (ROS) levels Inhibition of enzymatic activity
Kodedová et al. [115]	*Hortaea werneckii*	5,10,15,20-tetraphenylporphyrin associated with sulfonated polystyrene nanoparticles	395–630 nm, 23 W	Antifungal activity Reduction of fungal viability Increased ROS levels
Arriba et al. [24]	*Malassezia furfur*	Methyl aminolevulinate	PIT: 0 to 120 min 570–1400 nm, 200 mW/cm^2^, 30 min to 3 h	Reduction of fungal viability Biocompatibility Anti-inflammatory effects

## Data Availability

No new data were created or analyzed in this study. Data sharing is not applicable to this article.
